# Nurses' Trials—Martha and Mary

**Published:** 1887-02-19

**Authors:** 


### Words of Consolation.

[Communications for this column should be addressed, "Chaplain," care of the Editor of The Hospital, who will gratefully welcome any suggestions or inquiries from matrons, nurses, and the staff generally.]

### XXI.—Nurses' Trials—Martha and Mary.

It is most essential that nurses should maintain as high a standard as possible in devotion ; hard as they may find it at times to pray at all. It is essential for their own sakes, and for the sake of the sufferers whom they care for. Sympathy, cheerfulness, patience, gentleness, all the virtues in which nurses should excel, cannot be cultivated apart from communion with God. The most work, the best work, and the quietest work is done by those whose minds are stayed on Him.

To how many ministering unto Him in our hospitals may Jesus be saying : "Martha, Martha, thou art careful and troubled about many things : but one thing is needful : and Mary hath chosen that good part, which shall not be taken away from her." (Luke x, 41, 42.)

Martha is very busy and active, a most energetic, painstaking woman in her way. Mary finds time to sit at the feet of Jesus. But we do not suppose that Mary found this time at the sacrifice of duty, or our Lord would not have commended her as He did. "Martha, Martha." Our Lord's rebuke is very loving and gentle. He rebukes her not for her active service, but for being so full of care and trouble about it that she forgot for the time that anything else could be of greater importance. She was "over-anxious," for that is what the word "careful" really means. Only one thing should be the object of real and deep anxiety, and that "one thing" the good part Mary had chosen. These two sisters are often said to represent two very different classes of persons : those who serve Christ in busy active labour, and those who serve Him in secret adoration and devotion. This may be so. But we must not think our Lord desires us to draw a contrast between labour and devotion. His rebuke is for those who make light of devotion, or plead want of time for it, because they are called to work. The best and truest life is one (like that of angels) made up of both these forms of service—active labours and inward devotion (see S. P. C. K., Commentary on Luke x).

"There are in this loud stemming tide
Of human care and crime,
With whom the melodies abide
Of the everlasting chime ;
Who carry music in their heart
Through dusky lane and wrangling mart,
Plying their daily task with busier feet,
Because their secret souls a holy strain repeat."

*Christian Year.*

There are Marthas and Marys in nearly every hospital. If you ask which of the two render the best and most lasting service, we confess our preference for the latter. They may not seem to be so clever as their more talkative sisters, nor are they always hurrying to and fro quite at the same pace : but, as a rule, they are by far the most thoughtful. They are calm, collected, and peaceful about their work, possessing "the ornament of a meek and quiet spirit, which is in the sight of God of great price." The consequence is, they really get through a great deal more than they appear to do. Machines in perfect order, steam - engines, and many marvellous inventions in clockwork, when working at the highest pressure and producing the highest results, make the least noise ; yea, seem to be almost in repose. So also with the truest and ablest workers in the kingdom of heaven, so with those who combine Martha's energy with Mary's sweet repose. Already do they begin to exhibit in their lives here the mystery told of living creatures above : "They rest not day and night," and yet "They rest."

### AN ABLE CHAIRMAN.

In all public institutions, and especially in voluntary hospitals, the office of chairman is by no means a sinecure. Deemster Drinkwater, the chairman of the Isle of Man Hospital, however, when expressing the thanks of a public meeting the other day to the treasurer, secretary, and the committee generally, on being interrupted by one of the audience with the remark, "I think you leave yourself out," modestly replied, "his duties were easy to perform, but the gentlemen named, and the clergy, were the working bees in the hive." Here is sound evidence of an able chairman, faithfully discharging his duties with becoming but unusual modesty.

